# Identification of light-independent inhibition of human immunodeficiency virus-1 infection through bioguided fractionation of *Hypericum perforatum*

**DOI:** 10.1186/1743-422X-6-101

**Published:** 2009-07-13

**Authors:** Wendy Maury, Jason P Price, Melinda A Brindley, ChoonSeok Oh, Jeffrey D Neighbors, David F Wiemer, Nickolas Wills, Susan Carpenter, Cathy Hauck, Patricia Murphy, Mark P Widrlechner, Kathleen Delate, Ganesh Kumar, George A Kraus, Ludmila Rizshsky, Basil Nikolau

**Affiliations:** 1 Department of Microbiology, University of Iowa, Iowa City, IA 52242, USA; 2 Department of Chemistry, University of Iowa, Iowa City, IA 52242, USA; 3 Department of Microbiology, Immunology and Preventive Medicine, Iowa State University, Ames, IA 50011, USA; 4 Department of Food Science and Human Nutrition, Iowa State University, Ames, IA 50011, USA; 5 Department of Horticulture, Iowa State University, Ames, IA 50011, USA; 6 Department of Chemistry°, Iowa State University, Ames, IA 50011, USA; 7 Department of Biochemistry, Biophysics and Molecular Biology, Iowa State University, Ames, IA 50011, USA; 8 US Department of Agriculture-Agricultural Research Service, North Central Regional Plant Introduction Station, Ames, IA 50011, USA

## Abstract

**Background:**

Light-dependent activities against enveloped viruses in St. John's Wort (*Hypericum perforatum*) extracts have been extensively studied. In contrast, light-independent antiviral activity from this species has not been investigated.

**Results:**

Here, we identify the light-independent inhibition of human immunodeficiency virus-1 (HIV-1) by highly purified fractions of chloroform extracts of *H. perforatum*. Both cytotoxicity and antiviral activity were evident in initial chloroform extracts, but bioassay-guided fractionation produced fractions that inhibited HIV-1 with little to no cytotoxicity. Separation of these two biological activities has not been reported for constituents responsible for the light-dependent antiviral activities. Antiviral activity was associated with more polar subfractions. GC/MS analysis of the two most active subfractions identified 3-hydroxy lauric acid as predominant in one fraction and 3-hydroxy myristic acid as predominant in the other. Synthetic 3-hydroxy lauric acid inhibited HIV infectivity without cytotoxicity, suggesting that this modified fatty acid is likely responsible for observed antiviral activity present in that fraction. As production of 3-hydroxy fatty acids by plants remains controversial, *H. perforatum *seedlings were grown sterilely and evaluated for presence of 3-hydroxy fatty acids by GC/MS. Small quantities of some 3-hydroxy fatty acids were detected in sterile plants, whereas different 3-hydroxy fatty acids were detected in our chloroform extracts or field-grown material.

**Conclusion:**

Through bioguided fractionation, we have identified that 3-hydroxy lauric acid found in field grown *Hypericum perforatum *has anti-HIV activity. This novel anti-HIV activity can be potentially developed into inexpensive therapies, expanding the current arsenal of anti-retroviral agents.

## Background

The aerial parts of *Hypericum perforatum *L. (St. John's wort) have been used both historically and in modern times to treat various medical conditions, including depression, cancer, wounds, microbial infections and inflammation [1-9]. *Hypericum perforatum *is known to be rich in naphthodianthones, phloroglucinols and flavanoids [10]. Reported biological activities have been associated primarily with the naphthodianthones, hypericin and pseudohypericin, several flavanoids and the phloroglucinols, hyperforin and adhyperforin [1,11]. Recently, it has been suggested that synergy between the metabolic constituents is responsible for the anti-depressive activity of the extract [1].

The constituents hypericin and pseudohypericin effectively inhibit infection by a number of enveloped viruses of medical importance, including human immunodeficiency virus-1 (HIV), herpes simplex virus (HSV) and influenza A virus [12-15]. These inhibitory activities are light-dependent, which has lead to innovative strategies for delivering these compounds with bursts of light *in vivo *[16]; however, the need for light to activate hypericin remains problematic for many practical applications. Hypericin also has light-dependent cellular cytotoxicity [2,17,18]. Cytotoxicity may stem in part from hypericin eliciting rapid loss of mitochondrial potential [18]. Photocytotoxicity was responsible for the premature termination of a clinical trial that tested the efficacy of hypericin against HIV in AIDS patients [19].

Identification of additional anti-HIV therapies is needed as viral resistance to current drugs continues to develop. While botanicals have generally been a rich source of medicinal compounds, identification of botanically-based antivirals has been limited. Here, we sought to determine if light-independent anti-HIV activities are present in the constituent-rich species, *H. perforatum*. These studies were performed with chloroform extracts of aerial material, leading to the removal of most of the well characterized metabolites, including light-dependent hypericin [17]. Using bioactivity-driven fractionation of chloroform extracts, we were able to identify novel compounds having anti-HIV activity.

## Results

### Inhibition of HIV infection by chloroform extracts

Inhibition of HIV-1 infectivity by light-dependent constituents found in *H. perforatum *is well established, but these same compounds have extensive cellular cytotoxicity and require a light source for activation, thereby reducing their potential value as clinical antiviral therapeutics [16,20-22]. To determine if light-independent anti-HIV activity is also present in *H. perforatum *extracts, we began by extracting dried aerial material with chloroform. Chloroform was selected for the extraction solvent rather than ethanol as chloroform does not extract the light-dependent naphthodianthones or phloroglucinols [17,23]. Specifically, hypericin and pseudohypericin that were readily detected in ethanol extracts generated from the same plant samples were not detectable in these chloroform extracts [17,24].

**Figure 1:**
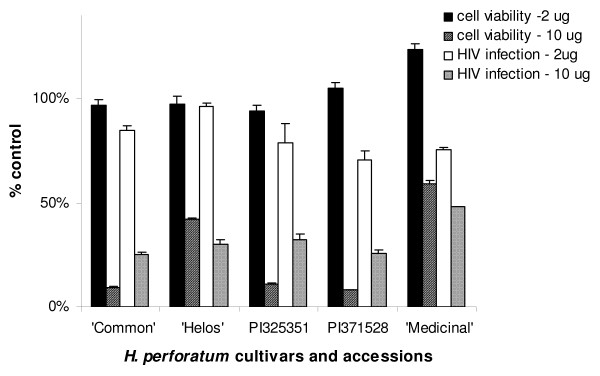
**HIV-1 infectivity inhibition and cellular cytotoxicity associated with chloroform extracts of accessions or commercial cultivars of *H. perforatum***. The HIV infectivity studies are represented as the ratio of HIV infectivity in the presence of extract divided by the infectivity in the absence of extract. The cytotoxicity is represented as the cell viability as detected in an ATPLite assay in the presence of extract divided by the viability of cultures in the absence of extract. All cells were exposed to equivalent concentrations of DMSO, the extract solvent. All studies were performed three times in triplicate and shown are means and standard error of the means. *p = 0.05.

As variation in constituent concentration and composition has been noted in different varieties of *H. perforatum *[25,26], we tested the ability of several accessions and commercial varieties to inhibit HIV infection (Fig. 1). Parallel cytotoxicity studies were performed to determine cell viability. Chloroform extracts of *H. perforatum *cultivars 'Common' and 'Medizinal' and accession PI 371528 had consistent, detectable inhibition against HIV at a concentration of 2 μg/ml. The impact of the extracts on cell viability was modest at these same concentrations. However, profound loss of cell viability was observed in wells treated with 10 μg/ml of extract and, while HIV infection was also inhibited at these higher concentrations, the loss of the cell monolayer is likely to be partially responsible for lower numbers of infected cells that were observed.

### Extract fractionation separated cytotoxicity from the anti-HIV activity

**Figure 2:**
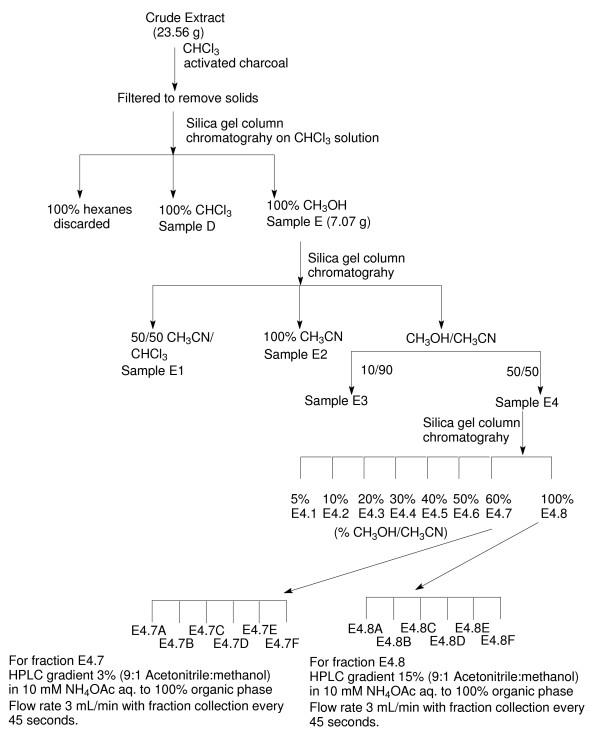
**Schematic of the fractionation protocol used to identify inhibitory activity against HIV-1**.

**Figure 3:**
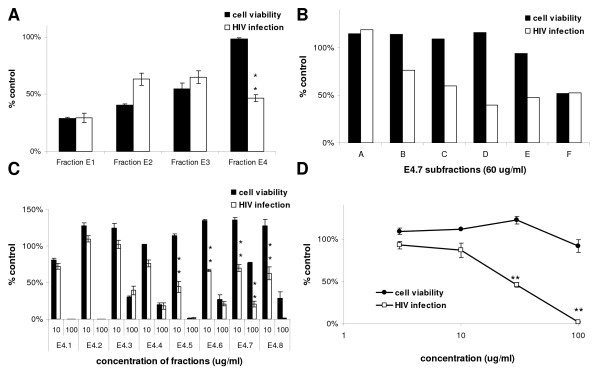
**Separation of constituents responsible for HIV infectivity inhibition and cytotoxicity**. A. Cell cytotoxicity and anti-HIV activity associated with fraction E subfractions. Studies were performed with 30 μg/ml of each E subfraction. Shown are results from a representative experiment. B. Activities of E4 subfractions. Subfraction E4.7 demonstrated significant anti-HIV activity with modest levels of cytotoxicity. All E4 subfractions were assessed for cytotoxicity and anti-HIV activity at 10 and 100 μg/ml. C-D. Activities of E4.7 subfractions. C. Anti-HIV activity and cytotoxicity of subfractions at a concentration of 60 μg/ml. D. Dose-response curve of subfraction E4.7d demonstrated anti-HIV activity in the absence of detectable cytotoxicity. All cells in all experiments were exposed to equivalent concentrations of DMSO, the extract solvent. The findings are shown as percent control values (the cytotoxicity or number of HIV antigen-positive cells in the presence of the various concentrations of the subfractions divided by the cytotoxicity or number of HIV antigen-positive cells in the absence of the compound). The statistical significance of HIV-1 inhibition was evaluated by comparing the inhibition of HIV infection to cytotoxicity at the same concentration of the subfraction. *p = 0.05; **p = 0.001.

Fractionation of the chloroform extract was undertaken to determine if the antiviral activity could be separated from the cytotoxic activity observed in the extracts. We selected *H. perforatum *'Common' for these fractionation studies. The extract initially was partially purified by column chromatography intended to generate groups of compounds to minimize the total number of bioassays necessary to identify active components. Initial fractions were assayed, active ones were further fractionated, and the cycle was repeated (Fig. 2). This process was guided by considering both cytotoxicity and inhibition of HIV infectivity. Evidence that separate constituents were responsible for these two biological activities was demonstrated following silica gel chromatography fractionation of fraction E using increasingly more polar solvents (Fig. 3a). The largest levels of cytotoxicity were observed in subfractions that were eluted under the most non-polar solvent conditions, whereas inhibition of HIV infectivity in the absence of cell cytotoxicity was evident in subfraction E4 that was eluted with a 1:1 mixture of acetonitrile/methanol.

Subsequent fractionation of E4 into eight subfractions was performed by using an initial elution solution of 5% methanol/95% acetonitrile to elute E4.1 evolving to 100% methanol that eluted E4.8. All subfractions were evaluated for antiviral activity and cytotoxicity at 10 and 100 μg/ml. In subfractions E4.5 through E4.8 at a concentration of 10 μg/ml, antiviral activity was observed with negligible loss of cell viability (Fig. 3b). At a concentration of 100 μg/ml, significant cytotoxicity was observed in all E4 subfractions; however, that observed in E4.7 was less than levels found in the others. The distribution of cytotoxicity across the elution gradient suggested that multiple compounds were present in the E4 subfraction that affected cell viability. Subfractions E4.7 and E4.8 were then subfractionated by using a reverse phase HPLC gradient, and six subfractions from each were collected and analyzed. Anti-HIV activity was lost during the E4.8 subfractionation (data not shown). However, anti-HIV activity was detected in subfractions E4.7b-e, and limited cytotoxicity was found in these subfractions (Fig. 3c). A dose-response curve from 3 to 100 μg/ml of E4.7d demonstrated antiviral activity with 50% inhibition of HIV (IC_50_) at a concentration of 27.6 μg/ml and an IC_90 _of 70.8 μg/ml (Fig. 3d). The E4.7d dose-response curve was performed in low light conditions under which the antiviral activity of any trace hypericin would not be activated (data not shown). Both the separation of antiviral activity from the cytotoxicity and the absence of light dependence of the antiviral activity argue that these fractions contain previously unidentified constituents that differ from known *H. perforatum *antiviral compounds such as hypericin.

### Analyses of compounds present in active fractions

**Figure 4:**
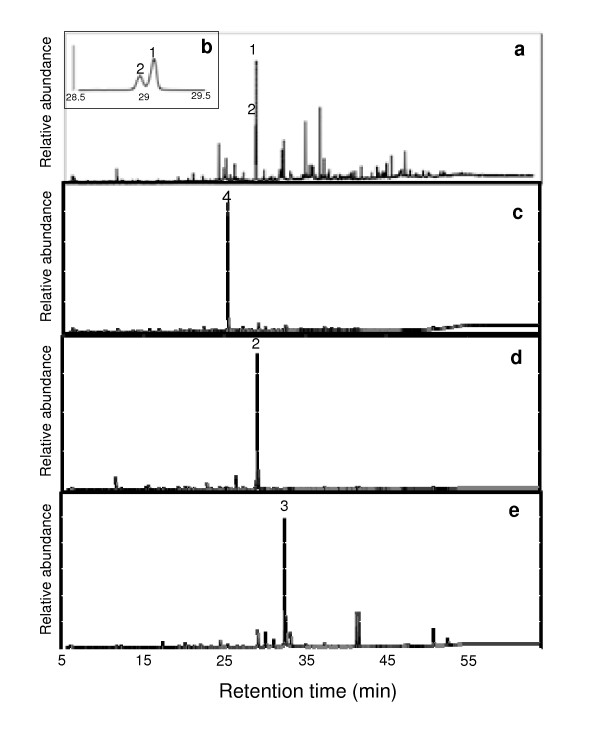
**GC analysis of bioactive fractions from *H. perforatum***. Total ion chromatograms of subfraction E4.7 (a) and expanded view at retention time 29 min (b), and subfractions derived from additional purification, E4.7c (c), E4.7d (d), and E4.7f (e). Peaks whose chemical identity was established by comparing their retention times and mass spectra to authentic standards (see Figure 5) are: palmitic acid (1), 3-hydroxy myristic acid (2), 3-hydroxy palmitic acid (3), and 3-hydroxy lauric acid (4).

**Figure 5:**
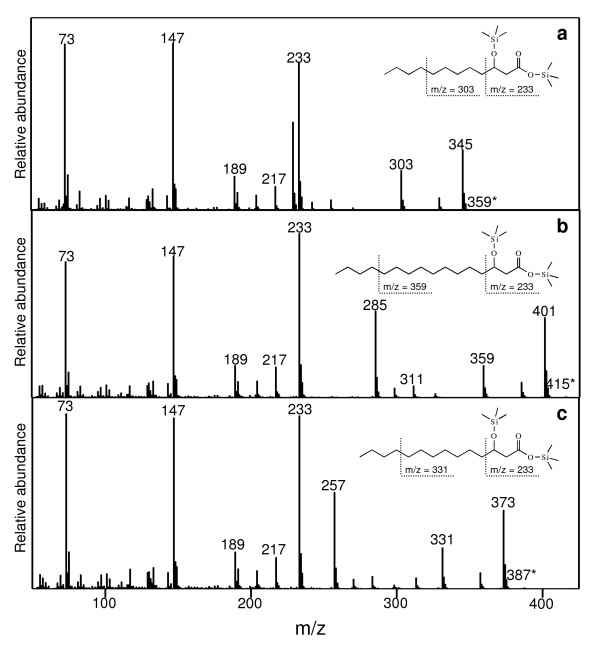
**Mass spectra of trimethylsiloxyl esters of peaksin *H. perforatum *extracts identified as 3-hydroxy lauric acid (a), 3-hydroxy palmitic acid (b), and 3-hydroxy myristic acid (c)**. The following ions indicate how to interpret these spectra: 1) the molecular ion (M+-1) is marked with an asterisk; 2) the abundant fragment at m/z = (M+-1)-15 is due to the fragmentation of the omega-methyl group; 3) the fragment ion at m/z = (M+-1)-57 is due to the left-most fragmentation indicated in each structure; and 4) the common fragment ion at m/z = 233 is due to the right-most fragmentation indicated in each structure.

To determine the chemical composition of the bioactive fraction E4.7, and two bioactive subfractions E4.7c and E4.7d, these samples were analyzed by using gas chromatography-mass spectrometry (GC-MS). As a control, subfraction E4.7f that contained cytotoxic activity, but no detectable antiviral activity, was analyzed in parallel. This analysis reveals that fraction E4.7 was a relatively complex mixture of metabolites (Fig. 4a), whereas all three subfractions were predominantly composed of a single metabolite that appeared to constitute about 80–90% of the detectable mass in each fraction (Fig. 4b–d). The chemical identity of these three major metabolites was determined based upon the fragmentation pattern obtained with mass-spectrometry (Fig. 5), and by comparing the chromatographic behavior of each metabolite with respect to authentic standards. These analyses identified the principal metabolites as 3-hydroxy lauric acid, 3-hydroxy myristic acids and 3-hydroxy palmitic acid in subfractions E4.7c, E4.7d and E4.7f, respectively (Fig. 4 and 5).

### Ability of a synthetic 3-hydroxy fatty acid to inhibit HIV infectivity

**Figure 6:**
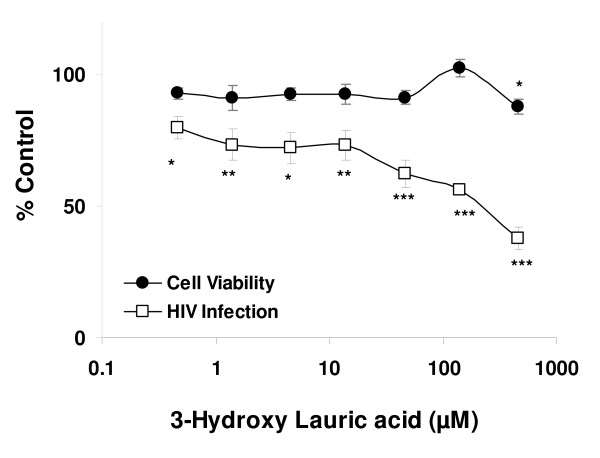
**Ability of synthetic 3-hydroxy lauric acid to inhibit HIV infectivity**. Increasing concentrations of 3-hydroxy lauric acid were incubated with HIV and added to HeLa37 cells. At 40 h following infection, cells were fixed and evaluated for HIV antigen staining. The cellular cytotoxicity of 3-hydroxy lauric acid was evaluated in parallel, in the absence of HIV. The findings are shown as percent control values (the cytotoxicity or number of HIV antigen-positive cells in the presence of the various concentrations of 3-hydroxy lauric acid divided by the cytotoxicity or number of HIV antigen-positive cells in the absence of the compound). Studies were performed three times in triplicate and shown are means and standard error of the means. *p = 0.05. **p = 0.005; ***p = 0.0001.

3-hydroxy lauric acid, the principal component of subfraction E4.7c, was synthesized. Evaluation of this compound in our HIV-inhibition assay demonstrated that relatively high concentrations (~10 μM and higher) of 3-hydroxy lauric acid inhibited HIV infectivity in a dose-dependent manner in the absence of detectable cytotoxicity (Fig. 6). The GC-MS analysis suggested that the concentration of 3-hydroxy lauric acid in fraction E4.7c was approximately 95 μM indicating that concentrations of this fatty acid in the subfraction were well within the range of active anti-HIV concentrations. These findings led us to conclude that it is likely that 3-hydroxy lauric acid present in E4.7c was at least partially responsible for the anti-HIV activity that we observed. The finding that our highly purified fractions were more inhibitory than pure 3-hydroxy lauric acid suggested the possibility that additional constituents present in the subfractions may contribute to the antiviral activity.

### 3-hydroxy-fatty acids can be detected in sterile *H. perforatum *seedlings

3-hydroxy fatty acids, such as 3-hydroxy lauric acid, 3-hydroxy myristic acid and 3-hydroxy palmitic acids, occur as natural intermediates of *de novo *fatty-acid biosynthesis, acylated to the phosphopantetheine prosthetic group of acyl-carrier protein (ACP). However, these hydroxylated fatty acids are not known to normally accumulate to high levels in plants. Rather the most abundant hydroxylated-fatty acids that accumulate to readily detectable levels in plants carry the hydroxyl group at the 2-position, the 9-position or the omega position of the acyl chain, and these are components of ceramides, cutin and suberin. On the other hand, such 3-hydroxy-fatty acids are a major component of Lipid A, a component of the lipopolysaccharide cell wall of gram-negative bacteria [27]. Although there have been many suggestions that Lipid A-like molecules may exist in plants [28], this is not a universally accepted concept. Therefore, it is formally possible that the 3-hydroxy-fatty acids that were recovered in subfractions E4.7c, E4.7d and E4.7f may in fact be extracted from bacteria present in the field-grown *H. perforatum *tissue, which was the starting material for the bioactivity-based fractionation.

**Figure 7:**
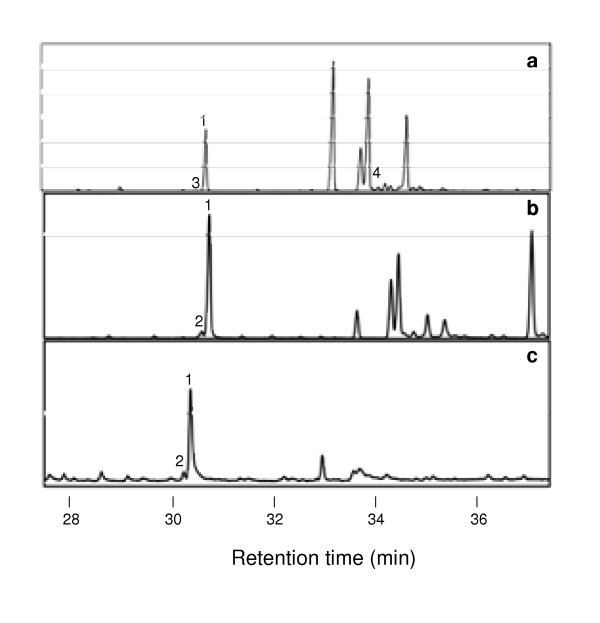
**GC analysis of fatty acid extracts prepared from aseptically grown seedlings (a), and dried, field-grown leaves (b) of *H. perforatum***. For comparison (c) shows the GC analysis of the original extract from which subfraction E4.7 was generated (see Figure 4). Fatty-acid peaks whose chemical identity was established by comparing their retention times and mass spectra to authentic standards are: palmitic acid (1), 3-hydroxy myristic acid (2), palmitelaidic acid (3), 3-hydroxy palmitic acid (4).

To test this possibility, we aseptically grew *H. perforatum *seedlings, and extracted and analyzed fatty acids from this sterile material. Fatty acids were extracted from 4-week-old sterile plants following the barium hydroxide hydrolysis of all acylated-lipids, and the recovered fatty acids were silylated and analyzed by GC-MS. For comparison, we also analyzed the fatty acids present in leaves from field-grown *H. perforatum *plants and fatty acids present in the original chloroform extract that was used to generate subfractions E4.7c and E4.7d. We were able to detect small quantities of 3-hydroxy myristic acid (Fig. 7, peak 2) in field-grown plant material (0.9 mole % of all detected fatty acids) and in the chloroform extract (0.3 mole % of all detected fatty acids), but this fatty acid was not detectable in the fatty acids extracted from *H. perforatum *seedlings grown under sterile conditions. Instead, we detected small quantities of 3-hydroxy palmitic acid in the sterile plants (Fig. 7, peak 4); however, this peak was not detected in the field-grown material. Thus, our analyses suggest that 3-hydroxy fatty acids are synthesized by *H. perforatum *and can be detected, but quantities of these fatty acids appear to be at the limit of detection in the initial extracts. Using our bioactivity-guided fractionation approach, we were able to identify subfractions that were predominately composed of these fatty acids. In addition, our findings suggest the possibility that growth conditions may influence the production of these fatty acids by *Hypericum*. These studies do not provide conclusive evidence as to the source of the 3-hydroxy fatty acids in our highly purified fractions, leaving open the possibility that either *H. perforatum *or gram-negative bacteria on the leaves of the plant are responsible for their production.

### Specificity of anti-HIV activity

We sought to determine the breadth of the antiviral activity we had found in the E4.7 and E4.8 subfractions. We tested them for antiviral activity against the distantly related lentivirus, equine infectious anemia virus (EIAV), in highly permissive equine dermis cells. No inhibition of EIAV infectivity was observed with addition of either E4.7 or E4.8 (Fig. 6). Higher doses of E4.7 were more cytotoxic to ED cells than that observed in our HIV studies in HeLa37 cells. These studies led us to conclude that these subfractions contain constituents including 3-hydroxy lauric acid that specifically target HIV and are not broadly inhibitory against other members of the lentiviral subfamily of retroviruses.

**Figure 8:**
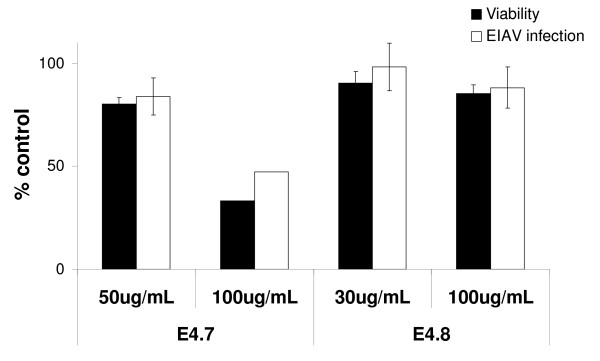
**The infectivity of the lentivirus equine infectious anemia virus (EIAV) is not inhibited by *H. perforatum *subfractions E4.7 or E4.8**. The subfractions were incubated with EIAV and added to ED cells (MOI = 0.005). Cultures were fixed at 40 h following infection and cells immunostained for EIAV antigens. Cytotoxicity of the subfractions on ED cells was performed in parallel, in the absence of EIAV. All data are shown as percent of control values.

## Discussion

Here we identify novel, light-independent anti-HIV activity in extracts generated from field-grown *H. perforatum*. This activity was found in chloroform extracts that do not contain the previously characterized light-dependent antiviral agents hypericin or pseudohypericin. Following extensive fractionation, we were able to separate the light-independent antiviral and cytotoxic activities, indicating that separate constituents are responsible for these activities. During the subfractionation process, the anti-HIV activity was found in the more polar fractions, whereas cytotoxic activities were distributed throughout the gradients. Upon GC/MS analysis of our most highly purified subfractions, two related 3-hydroxy-fatty acids, 3-hydroxy lauric acid and 3-hydroxy myristic acid, were found to be the most abundant compounds in the active subfractions E4.7c and E4.7d, respectively. These same fractions contained the most pronounced anti-HIV activity with minimal cytotoxicity. Synthetic 3-hydroxy lauric acid had significant inhibitory activity against HIV, suggesting that this compound was responsible for the antiviral activity observed in the E4.7c subfraction. Interestingly, subfraction E4.7f contained abundant 3-hydroxy palmitic acid. Substantial cytotoxicity was found in this subfraction and little or no anti-HIV activity was evident. This finding suggested that the length of the carbon chain impacts the biological activity of various 3-hydroxy fatty acids. Consistent with this possibility, shorter-chain 3-hydroxy fatty acids that were synthesized and tested in parallel with 3-hydroxy lauric acid were not found to contain anti-HIV activity (data not shown).

Synthesis and accumulation of 3-hydroxy fatty acids by plants has been suggested in the literature [28]. Lipid A that contains 3-hydroxy fatty acids was recently identified in green algae [29]. The angiosperm *Arabidopsis thaliana *is known to contain all of the genes required to synthesize Lipid A [30], suggesting that higher plants may also generate these fatty acids. In our studies, small quantities of 3-hydroxy myristic acid were detected in sterile seedlings, indicating that some 3-hydroxy fatty acids are indeed synthesized by *Hypericum *at least at some developmental stages. It is not clear how much of a contribution these endogenous 3-hydroxy fatty acids have towards the fatty acids found in our highly purified subfractions. Lipopolysaccharide from colonizing bacteria on field-grown plants may also be responsible for the presence of these fatty acids in our fractions.

3-hydroxy fatty acids have not been previously reported to have inhibitory activity against HIV. Furthermore, although these 3-hydroxy fatty acids comprise the fatty-acid chains of endotoxin from a number of human pathogens, individual, single-chain 3-hydroxy fatty acids bind poorly to the cellular protein MD-2 [31,32], do not elicit activation of macrophages through TLR4 [33,34] and are thought to contain little or no innate biological activity [33]. Here, we provide evidence that one or more of these 3-hydroxy fatty acids potentially can serve as a therapy against HIV. This inhibitory activity appears to be specific for HIV, as inhibition was not observed against the distantly related lentivirus, EIAV. Which step or steps within the HIV life cycle that are targeted by these fatty acids remain to be determined.

In addition to the anti-HIV activity, chloroform extracts also had significant cytotoxicity. Our studies suggest that there are multiple constituents present in the chloroform extracts that were responsible for this activity. For instance, subfractions of E4 that negatively affected cell viability eluted in a range of solvent conditions from 95% acetonitrile/5% methanol to 100% methanol. The broad range of solvent conditions that yielded fractions with cytotoxicity strongly implicates different constituents with varied polar characteristics. Reduced cell viability may be a result of induction of cell death and/or cessation of cell division. Clearly at high concentrations of the chloroform extracts, loss of cell viability was evident, since some extracts caused the loss of more than 90% of the cell monolayer. However, in some subfractions, such as E4.7, where cell viability was reduced by 20 to 30%, it is possible that the constituents had cytostatic activity. Future studies will be needed to determine which of these mechanisms is responsible for cytotoxicity and to identify specific constituents responsible for the activity.

Several light-independent cytotoxic constituents have been previously identified in *H. perforatum*. Hyperforin and procyanidin B2 have been shown to have cytotoxic activities in several cell lines [35,36]. However, neither of these compounds are found in chloroform extracts [23]. Additionally, uncharacterized *H. perforatum *lipophilic metabolites have been shown to have cytotoxic activities [23]. As lipophilic constituents might be expected to be present in chloroform extracts, these compounds may be responsible for some or all of the cytotoxicity observed.

In summary, we have successfully identified a new anti-HIV compound through bioguided fractionation of chloroform extracts of *Hypericum perforatum*. Our biological assay was sufficiently sensitive to allow detection of modest levels of antiviral activity that were present in the initial chloroform extract. Through the purification steps, the antiviral activity became readily apparent. Our findings implicate 3-hydroxy fatty acids in the antiviral activity. These may be endogenous to *Hypericum *or a result of bacterial growth on the field-grown plants.

## Materials and methods

### Growth of *H. perforatum *varieties and accessions

#### Plant Material

*Hypericum *field plots were established at the USDA-ARS North Central Regional Plant Introduction Station (NCRPIS) in Ames, Iowa. Three commercial cultivars and two unimproved populations of *Hypericum perforatum *were evaluated in this experiment. The 'Common' cultivar (Ames 28320, supplier's lot 16333) was obtained from Johnny's Selected Seeds (Winslow, ME), a seed company specializing in organic seeds, and the other cultivars, 'Helos' (Ames 27453, NCRPIS lot 04ncao01) and 'Medizinal' (Elixir™) (Ames 27452, NCRPIS lot 04ncao01), were grown from seeds supplied by Richter's Herb Specialists (Goodwood, ON, Canada). The cultivar 'Medizinal' was bred to contain a higher amount of napthodianthrones, and 'Helos' was bred for tolerance to anthracnose disease [6]. Two unimproved populations from the former Soviet Union, PI 325351 (NCRPIS lot 85ncab01) and PI 371528 (NCRPIS lot 75ncai01) were obtained from the NCRPIS, a public germplasm collection.

#### Plant Production

Seeds were germinated in petri dishes. After germination, seedlings were transferred to plastic trays (72 plugs/tray) containing Sunshine LC-1 Mix™(Sun-Gro Horticulture, Bellevue, WA). These seedlings were transplanted into field plots on 11 June 2003, at 118 days after seeding, at a plant height of 6 to 10 cm.

#### Plant Harvest

Material of 'Common' used in the fractionation studies was harvested on 23 July 2004, when plants were at 50% flowering. The studies that assessed the antiviral activity and cytotoxicity associated with chloroform extracts from several *H. perforatum *cultivars and accessions used plant material that was harvested on 16 June 2005, also at 50% flowering. Three plants per plot were harvested by cutting aerial parts 30.5 cm above the soil surface and placing them in mesh bags. Bags were placed in drying racks with forced air at 40°C for 8 days [37]. After the aerial parts were completely dry, dry weights were taken and tops were ground through a 40-mesh screen in a Wiley grinder.

### Extraction of *H. perforatum *aerial material

For the studies that investigated antiviral activity associated with the extracts, 6 g of dried plant material from each cultivar or accession was chloroform extracted and dried by rotary evaporation. For the fractionation studies, 450 g of ground aerial parts of *H. perforatum *'Common' were extracted with chloroform by Soxhlet extraction for 6 h. The extract was dried by rotary evaporation to yield a total of 64.93 g of material.

### Fractionation of chloroform extracts

The crude extract (23.6 g) was dissolved in CHCl_3 _(150 mL) and activated charcoal was added. After filtration, the filtrate was concentrated in vacuo, and the residue was placed on a short (~5 cm) silica column. Sequential elution with hexane (2 L), CHCl_3 _(4 L), and CH_3_OH (1 L) afforded three fractions that were concentrated in vacuo and subjected to bioassay. The active methanol fraction (7.1 g) was dissolved in 50:50 CH_3_CN:CH_3_OH and then subjected to column chromatography (3 cm, 180 cm^3 ^of silica). Sequential elution with 50:50 CH_3_CN:CHCl_3 _(600 mL), CH_3_CN (400 mL), 90:10 CH_3_CN:CH_3_OH (400 mL), 50:50 CH_3_CN:CH_3_OH (500 mL) gave four fractions (E1, 3.5 g; E2, 0.5 g; E3, 1.1 g; and E4, 2.0 g; ~100% recovery). After bioassay, the active fraction E4 (1.97 g) was subjected to column chromatography (3 cm, 100 cm^3 ^silica). The fraction was dissolved in 5 mL 95:5 CH_3_CN:CH_3_OH and eluted with a step gradient consisting of 400 mL each of (95:5, 90:10, 80:20, 70:30, 60:40, 50:50, 40:60, 0:100 CH_3_CN:CH_3_OH) affording eight new fractions (E4.1, 230 mg; E4.2, 240 mg; E4.3, 260 mg; E4.4, 240 mg; E4.5, 250 mg; E4.6, 140 mg; E4.7, 110 mg; E4.8, 90 mg; ~79% recovery). After bioassays revealed activity, samples E4.7 and E4.8 were further fractionated by reverse phase HPLC on a preparative scale C18 column. Samples E4.7 and E4.8 each were dissolved in 1:1:1:3 CH_3_CH_2_OH:CHCl_3:_H_2_O:CH_3_OH (3 mL), and purified by HPLC with a gradient elution (Solvent A. 10 mM aq NH_4_OC(O)CH_3_, Solvent B. 9:1 CH_3_CN:CH_3_OH). A gradient of 3% B:A to 100% B was used for sample E4.7, and a gradient of 15% B:A to 100% B was used for sample E4.8. The flow rate was set at 3 mL/min and tubes were collected every 45 sec. After concentration, 6 subfractions were obtained from each sample (E4.7a, 17 mg; E4.7b, 9 mg; E4.7c, 13 mg; E4.7d, 7 mg; E4.7e, 6 mg; E4.7f, 6 mg; ~54% total recovery) and (E4.8a, 34 mg; E4.8b, 8 mg; E4.8c, 3 mg, E4.8d, 4 mg; E4.8e, 4 mg; E4.8f, 4 mg; ~70% total recovery). These fractions were assayed as described above.

### Cell lines

HeLa37 cells were used for HIV studies [38]. This HeLa cell line expresses both CD4 and CXCR4 ectotopically and are permissive for HIV strains that use CCR5 or CXCR4 for entry. HeLa37 cells were maintained in high glucose DMEM with 10% fetal calf serum and pen/strep. Equine dermis cells (ED cells)(ATCC CCL57) used for the EIAV studies were also maintained in high glucose DMEM with 10% fetal calf serum and pen/strep.

### Generation of viral stocks

#### HIV

Stocks of HIV-1 were generated by transfecting a 150 cm plate of 80% confluent HEK 293T cells with 75 μg of the HIV molecular clone pNL4-3 by using the CaPO_4 _procedure [39]. Supernatants were collected at 48-h post-transfection, clarified to remove cell debris and frozen at -80°C until needed. Virus production was assessed by reverse transcriptase activity in the viral stocks and by the single round of infection assay in HeLa37 cells described below. RT assays were performed as previously described [40].

#### EIAV

Viral stocks of EIAV_MA-1 _were produced in ED cells. Supernatants were harvested from cells that were >95% positive for EIAV antigen as determined by EIAV antigen immunostaining. Supernatants were centrifuged for 5 min at 13,500 × g to remove cell debris, aliquoted, and frozen at -80°C until needed. Viral titers were determined by infection of ED cells by using the single round of infection assay described below.

### Viral-infection studies

#### HIV studies

All extracts or fractions were resuspended in DMSO. 2.5 × 10^2 ^infectious particles of HIV (MOI = 0.01) were combined with the concentrations of extracts or fractions noted in the figures. The amount of DMSO was adjusted so that equivalent concentrations of DMSO were used in all wells. No more than 0.5% DMSO was used, as HeLa37 cytotoxicity was observed at higher DMSO concentrations. The extract and HIV mixture was added to 2.5 × 10^4 ^cells/well of HeLa37 cells in a 48-well format. The cells were maintained for 40 h at 37°C in a CO_2 _incubator. Cells were fixed in 75% acetone/25% water and immunostained for HIV antigens with human anti-HIV antisera (1:500) followed by HRP-conjugated goat anti-human IgG (1:500). 3-amino-9ethyl-carbazole was used as the horse radish peroxidase substrate. Plates were dried and wells were counted for the number of HIV antigen-positive cells. Numbers of HIV antigen-positive cells in the presence of extract, fraction or fatty acid were divided by the number of HIV antigen-positive cells present in control wells that did not contain extracts, and these values are expressed as % control.

#### EIAV studies

All studies were performed in ED cells. All extracts or fractions were resuspended in DMSO. 2.5 × 10^2 ^infectious particles of EIAV were combined with the concentrations of extracts or fractions noted in each experiment. The amount of DMSO was adjusted so that equivalent concentrations of DMSO were used in all wells. No more than 1% DMSO was used, as ED cell cytotoxicity was observed at higher DMSO concentrations. The extract and virus mixture was added to 5 × 10^4 ^cells/well of ED cells in a 48-well format to yield a MOI of ~0.005. The infections were maintained for 40 h. Cells were fixed with75% acetone/25% water at 40 h following initiation of the infection, and anti-EIAV immunostaining of the cells was performed as previously described [41]. The EIAV antigen-positive cells within the monolayer were enumerated. Numbers of EIAV antigen-positive cells in the presence of the fractions were divided by the number of EIAV antigen-positive cells present in control wells that did not contain extracts, and these values were expressed as % control.

### Cell-viability studies

ED or HeLa37 cells were plated and treated with extracts, fractions or fatty acid as described above. Cell viability was monitored at 40 h after treatment initiation by ATPLite Assay (Packard Biosciences) per manufacturer's instructions.

### Aseptic growth of *H. perforatum *seedlings

*Hypericum perforatum *(Accession Ames 28320, lot 06ncao01) seeds were surface sterilized by treating for 7 min with a solution consisting of 50% (v/v) Bleach and 0.05% (v/v) TritonX-100. After washing the seeds 3 times with sterile water, the seeds were placed on sterile wet 3 MM Whatman paper filters in sterile Petri plates. After germination, seedlings were aseptically transferred to individual Magenta boxes containing 25 ml of sterile 1% agar prepared in 1× Murashige & Skoog Basal Medium containing Gamborg Vitamins with macro and micronutrients (PhytoTechnology lab) Boxes were placed in a growth room maintained at 21°C, and under a 16-h light cycle, illuminated at 50 mmol m^-2 ^s^-1^.

### Lipid extraction

Lipid-bound fatty acids were extracted by a modification of a previously published method [42]. Approximately 0.1 g fresh weight of aerial tissue or 0.05 g of root tissue, from 4-week-old *H. perforatum *plants, spiked with a known quantity of nonadecanoic acid as an internal standard, was homogenized with 1 mL of 10% (v/v) barium hydroxide and 0.55 mL of 1,4-dioxane, and the mixture was heated at 100°C for 24 h. After acidification with 6 M hydrochloric acid, fatty acids were extracted with two aliquots of hexane, which were pooled and taken to dryness under a stream of N_2 _gas.

### Derivatization and GC/MS analysis

All samples were silylated [42,43] by dissolving the dried extracts in 1 mL of acetonitrile, and adjusted to 6% of bis-trimethylsilyl-trifluoroacetamide and 10% trimethyl-chlorosilane. Samples were incubated at 65°C for 20 min, cooled, and filtered through a polytetrafluoroethylene filter. Silylated samples were analyzed by using an Agilent GC series 6890 equipped with an HP-5ms capillary column (30 m × 0.32 μm, inner diameter) using helium as the carrier gas. The GC was coupled to an Agilent 5973 mass detector. The injector was held at 250°C, the oven was initially at 70°C for 4 min, then ramped at 5°C/min to 320°C and held at that temperature for 6 min. Resulting chromatograms were integrated with Agilent's HP enhanced ChemStation TM G14701 BA version D.02.00.275.software. Peaks were identified by comparing acquired mass spectra with the Agilent NIST05 mass spectrum library.

### Synthesis of hydroxy fatty acids

^(1) ^To a solution of diisopropyl amine (3.3 mL, 24 mmol) in THF (20 mL) at 0°C, *n*-BuLi was added (8.8 mL, 2.5 M solution in hexane). The solution was cooled to -78°C with stirring, and then acetic acid (0.6 g, 10 mmol) in 5 mL of THF was added. After 30 min, decanal (0.56 g, 10 mmol) in 5 mL of THF was added. The mixture was stirred for 1 h and then brought to RT slowly. The reaction was diluted with dichloromethane and washed with ammonium chloride solution and the layers then were separated. The organic layer was dried with sodium sulfate and then concentrated. The solid mass was crystallized from dichloromethane to give 3-hydroxydodecanoic acid (mp 141°C). This compound has previously been prepared from the beta-keto ester [44].

^1^H NMR (400 MHz, CDCl_3_) δ 4.05 (m, 1H), 2.65 – 2.48 (m, 2H), 1.59 – 1.26 (m, 16H), 0.89 – 0.85 (t, J = 6.6 Hz, 3H)

### Statistical analysis

All studies were performed at least three independent times except where noted in the figure legends. Means and standard errors of the mean are shown. To obtain IC_50 _and IC_90 _values for dose response curve data, the results were evaluated in the software Table Curve by using a best fit logistic dose response curve equation. Student's t-test was used to evaluate the statistical differences between treatments, utilizing the two-tailed distribution and two-sample equal variance conditions. P-values were accessed by comparing the level of infectivity with treatment to the level of cytoxicity seen with that treatment. A significant difference was determined by a p-value of < 0.05, and significance levels were identified in each figure. If the p-value was > 0.05, the data were not considered significantly different.

## Abbreviations

HIV: human immunodeficiency virus; EIAV: equine infectious anemia virus; IC_50_: inhibitory concentration 50 (concentration of compound that inhibits 50% of virus infectivity); IC_90_: inhibitory concentration 90 (concentration of compound that inhibits 90% of virus infectivity); DMSO: dimethyl sulfoxide; ED cells: equine dermis cells; MOI: multiplicity of infection.

## Competing interests

The authors declare that they have no competing interests.

## Authors' contributions

WM was responsible for oversight of the project including design and coordination of the study. In addition, WM wrote the manuscript and generated final versions of the figures. JPP and CSO were responsible for all of the HIV studies that were performed. MAB was responsible for the EIAV studies that were performed. MPW and KD were responsible for the oversight of growth of the *Hypericum *and participated in the harvesting and processing of the plant material. PM and CH were responsible for the production of the *Hypericum *chloroform extracts. JN and DW were responsible for all fractionation of the chloroform extract. LR and BN were responsible for GC/MS and LC/MS analysis of the fractions and sub-fractions. In addition, they were responsible for all studies with sterile *Hypericum*. SC and NW were responsible for initial studies with *Hypericum *chloroform extracts and identification of light-independent antiviral activity. GK and GAK were responsible for the synthesis of all pure fatty acids evaluated. All authors read and approved the final manuscript.
